# Treadmill Training Improves Overground Walking Economy in Parkinson’s Disease: A Randomized, Controlled Pilot Study

**DOI:** 10.3389/fneur.2014.00191

**Published:** 2014-09-26

**Authors:** Miguel Angel Fernández-del-Olmo, Jose Andres Sanchez, Olalla Bello, Virginia Lopez-Alonso, Gonzalo Márquez, Luis Morenilla, Xabier Castro, Manolo Giraldez, Diego Santos-García

**Affiliations:** ^1^Faculty of Sciences of Sport and Physical Education, Department of Physical Education, University of A Coruña, A Coruña, Spain; ^2^Physical Therapy Department, University School of Physical Therapy, University of A Coruña, A Coruña, Spain; ^3^Department of Neurology, Hospital A. Marcide, Ferrol, Spain

**Keywords:** Parkinson’s disease, treadmill, gait, exercise, walking economy

## Abstract

Gait disturbances are one of the principal and most incapacitating symptoms of Parkinson’s disease (PD). In addition, walking economy is impaired in PD patients and could contribute to excess fatigue in this population. An important number of studies have shown that treadmill training can improve kinematic parameters in PD patients. However, the effects of treadmill and overground walking on the walking economy remain unknown. The goal of this study was to explore the walking economy changes in response to a treadmill and an overground training program, as well as the differences in the walking economy during treadmill and overground walking. Twenty-two mild PD patients were randomly assigned to a treadmill or overground training group. The training program consisted of 5 weeks (3 sessions/week). We evaluated the energy expenditure of overground walking, before and after each of the training programs. The energy expenditure of treadmill walking (before the program) was also evaluated. The treadmill, but not the overground training program, lead to an improvement in the walking economy (the rate of oxygen consumed per distance during overground walking at a preferred speed) in PD patients. In addition, walking on a treadmill required more energy expenditure compared with overground walking at the same speed. This study provides evidence that in mild PD patients, treadmill training is more beneficial compared with that of walking overground, leading to a greater improvement in the walking economy. This finding is of clinical importance for the therapeutic administration of exercise in PD.

## Introduction

Gait disorders are common symptoms of Parkinson’s disease (PD) patients (1). PD gait is characterized by a particular difficulty with the internal regulation of stride length (2). Associated disturbances such as forward-flexed trunk, postural instability, and high stride-to-stride variability, are also common in this population (3, 4). In addition, the walking economy, defined as the rate of oxygen consumed per distance during walking, is less efficient in PD patients compared with healthy subjects and may contribute to the excess fatigue often observed in this population (5). Therefore, gait performance represents one of the major factors in determining the independence and quality of life of the patients (6) and thus, one of the main focuses of physical rehabilitation is to improve the gait deficits in PD.

In the last decade, a growing number of studies have been conducted in order to explore the impact of exercise on PD symptoms (7). Among those studies, the use of treadmill has emerged as an important tool to improve the gait performance in PD patients. Gait improvements have been reported as a result of treadmill training programs with different interventions and conditions (8–10). A recent study showed that lower-intensity treadmill exercise resulted in greater improvements in gait speed in comparison with a higher-intensity treadmill exercise (11). Another recent study from our group comparing two walking training programs, one performed on a treadmill and another overground, showed that only the former lead to an improvement in the stride length at the preferred and maximal speed (12). Thus, these findings suggest that the treadmill has a specific therapeutic effect in PD patients.

Besides the abovementioned benefits, the effects of treadmill training on cardiopulmonary parameters in PD patients have not been extensively investigated. Only three studies have measured oxygen uptake by indirect calorimetry, reporting that treadmill training can improve the walking economy in PD patients (11, 13, 14). However, the cardiopulmonary parameters were obtained from subjects while walking on the treadmill and thus, could reflect a specific improvement during treadmill walking rather than a more generalized and functional effect of overground walking.

Therefore, the main goal of this study was twofold: (i) to explore the effects of treadmill and overground walking training programs on the overground walking economy of PD patients; (ii) to explore the metabolic differences between walking on a treadmill and overground, since this relationship has not been described in PD patients. These would be of relevance to determine whether treadmill training may be prescribed as a potential therapy for reduction of fatigue associated with daily activities in PD.

## Materials and Methods

### Patients

Twenty-two patients with mild PD (13 males and 9 females, mean age ± SD 58.72 ± 10.35 years) were recruited from the local community and local PD Association, under the supervision of a neurologist. Nineteen patients were in a mild state (range of H&Y from I to II) and three in a moderate state (III of H&Y). PD patients were excluded if they had a past history of neurological conditions other than PD, orthopedic, or visual disturbance that affected walking ability. A treadmill graded exercise test (with monitoring of ECG and blood pressure) was conducted to detect any signs of cardiovascular or autonomic dysfunction. A fundamental requirement for inclusion in the study was the ability to walk for 10 min without stopping, walking aids, or assistance. All tests were carried out while the patients were ON medication, corroborated by a neurologist and consistently done at the same time of day for each patient. The level of functional disability was determined using the Unified Parkinson’s Disease Rating Scale (UPDRS) and the Hoehn and Yahr scale (H&Y). No patient showed dementia as assessed by a mini-mental state examination (MMSE >26) and all of the patients provided their written informed consent according to the declaration of Helsinki (1964), before entering the study. The local ethic committee of University of A Coruña approved the experimental protocol. Details of the subjects are shown in Table 1.

**Table 1:** **Details of Parkinson’s disease patient characteristics**.

Patient number	Age (years)	Sex	Height (m)	Disease duration (years)	Type	H&Y	UPDRS motor	Medication per day (mg)
**TREADMILL TRAINING PATIENTS**								
1	66	F	1.60	12	T	2.5	12	Levodopa/carbidopa 200/50, levodopa/benserazide 550/137.5, rotigotine 6, rasagiline 1, amantadine 200
2	79	F	1.49	7	AR	3	35	Levodopa/carbidopa 400/50, pramipexole 0.18
3	58	M	1.76	6	M	2	23	Levodopa/carbidopa 500/125, rasagiline 1, rotigotine 4
4	60	M	1.69	6	AR	2	12	Levodopa/carbidopa 800/200, entacapone 800, pramipexole 3.15
5	60	M	1.68	7	M	2	13	Levodopa/carbidopa 500/125, ropinirole 12, trihexyphenidyl 2
6	62	F	1.59	2	AR	3	31	Levodopa/carbidopa 600/150, entacapone 600, rotigotine 6, pramipexole 3.15
7	68	M	1.65	1	AR	2.5	16	Levodopa/carbidopa 150/37.5, entacapone 600, rasagiline 1
8	50	M	1.70	5	AR	2	19	Levodopa/carbidopa 300/75, rasagiline 1, rotigotine 8
9	67	M	1.80	3	M	2	15	Levodopa/benserazide 500/125, rasagiline 1, pramipexole 2.64
10	39	F	1.63	1	T	2	18	Levodopa/carbidopa 375/93.75, entacapone 600, rasagiline 1
11	45	M	1.74	3	M	2	11	Levodopa/carbidopa 225/56.25, entacapone 600, rasagiline 1, rotigotine 4
Mean	59.45		1.66	4.82		2.27	18.64	
SD	11.32		0.08	3.28		0.41	7.99	
**OVERGROUND TRAINING PATIENTS**								
1	55	M	1.72	6	M	2	23	Levodopa/carbidopa 225/56.25, entacapone 600, rasagiline 1, ropinirole 20
2	56	M	1.65	2	AR	1.5	12	Pramipexole 2.1
3	51	F	1.68	5	AR	2	36	Levodopa/benserazide 600/150
4	46	F	1.61	6	M	2.5	23	Levodopa/carbidopa 150/37.5, entacapone 600, rasagiline 1
5	46	F	1.62	1	AR	2	23	Levodopa/carbidopa 150/37.5, rasagiline 1, pramipexole 3.15
6	62	M	1.73	4	AR	2	12	Levodopa/carbidopa 150/37.5, entacapone 600, pramipexole 3.15
7	61	F	1.57	6	M	2	23	Levodopa/carbidopa 500/50, pramipexole 3.15
8	63	M	1.74	8	AR	2	21	Levodopa/carbidopa 750/187.5, entacapone 1000, rasagiline 1, pramipexole 3.15
9	78	M	1.62	9	AR	3	38	Levodopa/carbidopa 400/100, levodopa/benserazide 700/175, ropinirole 20
10	54	F	1.57	6	AR	2.5	25	Levodopa/carbidopa 400/100, entacapone 800, rotigotine 12
11	66	M	1.67	1.5	T	1	7	Levodopa/carbidopa 375/37.5, rasagiline 1
Mean	58.00		1.65	4.95		2.05	22.09	
SD	9.38		0.06	2.59		0.52	9.44	
*p* Value between groups	0.74		0.63	0.91		0.42	0.36	

### Procedure

The patients were randomly assigned to a treadmill training group (Gtreadmill) or an overground training group (Gground). Before the start of the training programs (T0), the patients performed the following tests in this particular order: (i) walking overground for 6 min at their preferred speed; (ii) walking on a treadmill for 6 min at their preferred speed (the same speed that was obtained for overground walking). A minimum of 5 min rest was required between tests. The walking overground test was again evaluated a week after the cessation of the training program (T1).

### Training programs

The training program consisted of 5 weeks, three session/week of walking on a treadmill or walking overground. In the first week, each session consisted of four bouts of 4 min of walking, with 3 min rest between bouts. Each week, an additional 4 min was added. The walking speed during the training sessions remained constant and was determined as the individual overground preferred speed obtained for each subject during the first evaluation. Patients from the Gtreadmill group were asked to walk on a treadmill (SporsArts 6300, Sports Arts Fitness) without body weight support, wearing a safety harness to prevent falls. In addition, all patients were asked to hold on to the handrails of the treadmill regardless of whether they needed to or not. All patients were able to walk on the treadmill at their overground comfortable speed from the first block of the first session. The training of the Gground group was conducted in an indoor facility 60 m long and 10 m wide. In order to control the walking speed of the Gground patients, each patient wore an MP3 device that provided auditory cues. Between each auditory cue, the patients had to walk a distance of 20 m. To provide feedback to the patients regarding their speed, cones were located on the side of the walkway each 20 m. At the moment of each auditory cue, the patient had to arrive to the cone. The pace of the auditory cues was adjusted to the overground preferred speed of each patient. In a pilot study, we determined that the auditory cues did not affect any of the gait parameters in the patients. The walking speed of each patient was monitored across each training sessions in order to confirm that the patient was walking at the desired speed. During the period of training, the patients did not change their daily activities or medication.

### Gait evaluation tests

The overground walking test was conducted in an athletic indoor facility 60 m long and 10 m wide. Subjects were required to walk at their preferred speed on a 40-m course marked by cones at each end. The cones at each end were placed in a semi-circumference of 10 m of length to allow the subjects to walk in a continuous loop until directed to stop.

The treadmill walking tests were conducted without body weight support and subjects wore a safety harness to prevent falls. All patients were asked to hold on to the handrails of the treadmill regardless of whether they needed to or not.

### Data acquisition

Kinematic gait parameters were recorded (1 GHz) using foot-switches (0.5″ Force Sensing Resistors, Interlink Electronics, USA) placed under the heel and toe of each foot. The gain of foot-switches was adjusted to the subject’s weight. The Wi-Fi acquisition unit (BTS PocketEMG, BTS Spa, Italy), weighing <300 g, was worn on the waist. During the overground walking test, the foot-switches were synchronized with photocells (Omron E3G-R17, Omron Corporation, Japan) positioned at each end of the 40 m line. An operator controlled the online data acquisition. Off-line analysis was performed using BTS MyolabClinic software (BTS Spa, Italy). The variables measured for each condition of gait included: speed (meter/second); stride length (meter); cadence (steps/minute); and stride time variability (%). For the overground walking test, the turnings were excluded from the analysis.

Walking economy was assessed via indirect calorimetry (Cosmed k4b2, Cosmed, Italy) for both overground and treadmill gait tests. Oxygen consumption and carbon dioxide production were continuously collected and analyzed with breath-by-breath measurement, and averaged over 15 s intervals to reduce variability. Walking energy expenditure was defined as the average volume of oxygen consumed (milliliter per kilogram per minute) during the last 3 min of each tests. To provide a standardized measure of the metabolic cost during the gait, walking energy expenditure was divided by the speed to derive energy expenditure per meter (milliliter per kilogram per meter). Respiratory exchange ratio (RER) (CO_2_ production/O_2_ uptake) was also calculated to indirectly determine the relative contribution of carbohydrate and lipids to the overall energy expenditure. Heart rate (HR) was monitored during exercise tests with a telemetric HR monitor (Polar RS800CX; Finland).

### Data analysis

To determine differences between overground walking and treadmill walking paired students *t*-tests were conducted for the metabolic and kinematic parameters of the total sample (*n* = 22).

To compare the cardiovascular effects of the two training programs, ANOVA of repeated measures was conducted for the overground walking metabolic parameters with Group (Gtreadmill and Gground) and Time (T0, and T1) as factors.

## Results

**Figure 1:**
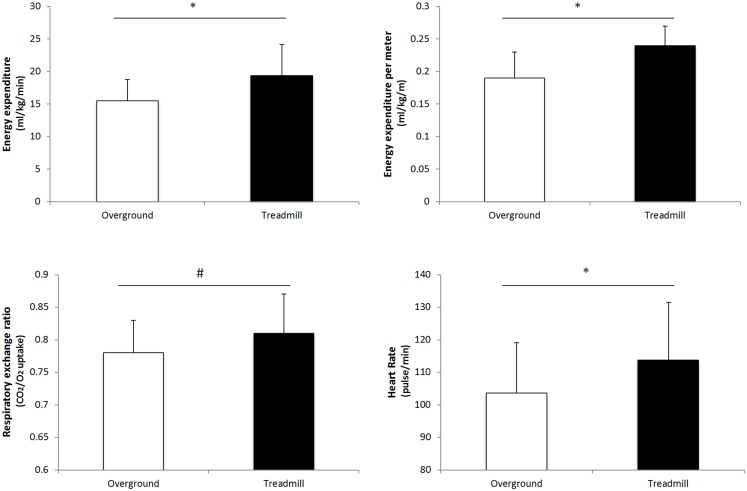
**Comparison of metabolic parameters for overground vs. treadmill walking**. *p < 0.001; ^#^*p* = 0.001.

The comparison between walking overground and walking on a treadmill showed a significantly higher walking energy expenditure (milliliter per kilogram per minute) and expenditure per meter (milliliter per kilogram per meter) for treadmill walking compared with overground walking (*t* = 5.61, *p* < 0.001; *t* = 5.96, *p* = < 0.001, respectively). In addition, RER and HR were also significantly higher walking on a treadmill than walking overground (*t* = 3.78, *p* = 0.001; *t* = 5.57, *p* = < 0.001, respectively) (Figure 1). There were no differences in the kinematic parameters between overground and treadmill walking (Table 2).

**Table 2:** **Comparison of kinematic parameters for overground vs. treadmill walking**.

	Overground	Treadmill	*t*	*p*
Speed (m/s)	1.30 ± 0.27	1.30 ± 0.27		
Cadence (steps/min)	118.28 ± 10.26	117 ± 12.59	0.43	0.67
Stride length (m)	1.31 ± 0.21	1.32 ± 0.21	0.60	0.53
Stride time variability (%)	1.84 ± 1.05	1.98 ± 1.08	0.88	0.38
Energy expenditure (ml/kg/min)	15.54 ± 3.24	19.40 ± 4.78	5.61	<0.001
Energy expenditure per meter (ml/kg/m)	0.19 ± 0.04	0.24 ± 0.03	5.96	<0.001
Respiratory exchange ratio (CO_2_/O_2_ uptake)	0.78 ± 0.05	0.81 ± 0.06	3.78	0.001
Heart rate (pulse/min)	103.6 ± 15.52	113.75 ± 17.77	5.57	<0.001

The analysis of the metabolic effects of the two training programs on the overground walking test (Table 3) showed a significant Group*Time interaction for the energy expenditure per meter [*F*_1,20_ = 5.48 *p* = 0.03; η^2^ = 0.24; observed power (OP) = 60%], but no significant Group and time main effects. *Post hoc* analysis indicated that the Gtreadmill group decreased significantly the energy expenditure per meter after the treadmill training program (*p* = 0.04). No changes in the energy expenditure per meter were reported for the Goverground group. The ANOVAs of the energy expenditure, RER, and HR did not show significant main effects or interactions.

**Table 3:** **Metabolic effects on the evaluated tasks**.

		Gground		Gtreadmill	
		T0	T1	T0	T1
Walking at preferred speed	Energy expenditure (ml/kg/min)	14.81 ± 3.87	16.19 ± 4.53	15.62 ± 2.26	15.89 ± 3.07
	Energy expenditure per meter (ml/kg/m)	0.18 ± 0.02	0.19 ± 0.031	0.21 ± 0.04	0.19 ± 0.02*
	Respiratory exchange ratio (CO2/O2 uptake)	0.77 ± 0.06	0.78 ± 0.06	0.78 ± 0.05	0.76 ± 0.05
	Heart rate (pulse/min)	98.6 ± 18.45	95.59 ± 17.5	104.22 ± 11.79	104.63 ± 16.29
Walking on treadmill at initial preferred speed	Energy expenditure (ml/kg/min)	19.12 ± 3.97		19.60 ± 3.36	
	Energy expenditure per meter (ml/kg/m)	0.23 ± 0.02		0.25 ± 0.04	
	Respiratory exchange ratio (CO2/O2 uptake)	0.80 ± 0.06		0.82 ± 0.07	
	Heart rate (pulse/min)	107.95 ± 18.18		117.04 ± 18.16	

*p < 0.05.

## Discussion

The main finding of our study is that 5 weeks of low intensity treadmill training improved the walking economy in PD patients. After the treadmill program, patients showed an improved walking economy when walking overground at their preferred speed. This improvement was not observed in patients that trained overground. In addition, we found that walking on a treadmill at the preferred overground speed seems to demand higher metabolic cost compared with overground walking.

### Energy cost overground vs. treadmill walking

Our findings showed that when PD patients walked at the same speed overground and on a treadmill, the energy cost was greater during the latter condition, as demonstrated by higher metabolic parameters such as energy expenditure, energy expenditure per meter, RER, and HC. To our knowledge, this is the first study to compare the energy cost of overground vs. treadmill walking in PD patients. Our results are in line with previous studies conducted in healthy elderly subjects (15, 16). In our study, the higher metabolic cost on the treadmill is unlikely to be due to changes in the kinematics parameters since these did not differ between the overground and treadmill walking conditions. The stride length and cadence remained unaffected for the two gait conditions. Although stride length increases cadence reductions in cadence have been reported for treadmill vs. overground walking in PD patients (17), those changes were limited to patients with a more advanced degree of disease severity (III in the H&Y scale). Therefore, it remains to be explored whether the metabolic effects observed in our study can be replicated in more severe PD patients.

The higher energy cost observed in PD patients walking on a treadmill may be due to greater muscular activation in comparison with overground walking, although no studies to date have compared the EMG pattern between these conditions. In healthy subjects, the treadmill generally induces greater EMG amplitudes of lower-limb muscles (18, 19), probably as a result of a greater balance demands during treadmill walking (20). This change in EMG may reflect a higher agonist and antagonist activation (co-activation), leading to improved balance and stability on the treadmill, but resulting in a higher metabolic cost. Moreover, it has been reported that age-related adaptations in the recruitment pattern of leg muscles during gait significantly contributes to the higher metabolic profile in the elderly (21).

It is noteworthy that, in our study, all the patients were requested to use the handrails during the treadmill walking. In healthy subjects, the use of handrail leads to a decrease in oxygen consumption and HR at a given workload (22, 23). If those findings also apply to PD patients, then we would expect to find even greater differences in the metabolic parameters between the treadmill and overground conditions if subjects were allowed to walk with a free arm swing.

We must point out that the patients always walked on the ground first followed by the treadmill walking. The reason for which we did not randomize or counterbalance the order of the conditions was to avoid a treadmill generalization effect, where subjects tend to walk faster and with higher cadence overground after walking on a treadmill, even when asked to walk at their preferred speed (17). The absence of a counterbalance order could add a certain degree of fatigue to the treadmill walking test leading to an overestimation of its metabolic cost. However, this is unlikely since the HR was monitored across the session to ensure that the initial HR was similar at the beginning of both walking tests. In addition, the patients remained seated after the overground walking test for a minimum of 5 min regardless of their HR values.

To summarize so far, our study shows that walking on a treadmill, at overground preferred speed, demands more energy than walking overground even though the kinematic parameters remain unchanged.

### Metabolic effects of training programs

The most relevant finding of the current study is that a low volume and intensity walking exercise program performed on a treadmill leads to an improvement in the efficiency of PD patients to walk overground at their preferred speed, as indicated by a reduction in the walking energy expenditure. In a previous report, we showed that the Gtreadmill group increased their preferred speed and stride length after the training program (12). Thus, after the treadmill training program, the patients were not only able to walk faster and with longer stride length but also with lower energy expenditure. This finding is of relevance for several reasons:

(i) Although, previous studies have reported that treadmill training can improve the walking economy in PD patients, this effect was tested during treadmill walking (6, 13, 14) and may be specific to this condition. To our knowledge, this is the first randomized study that measured the effects of two exercise programs (overground vs. treadmill walking) on the walking energy cost in PD patients. Thus, we provide the first evidence that the cardiovascular effects of a treadmill training program can be generalized to a daily activity such as overground walking.
(ii) The intensity (speed) and volume (minutes) of the treadmill training program in our study are so far the lowest from those reported in previous studies (11, 13, 14, 24–28) but enough to improve both kinematic (12) and metabolic parameters. These results support the notion that even low intensity exercise can benefit PD patients, as opposed to high intensity training, which can cause fatigue.

As discussed, the straightforward explanation for the improvement in the walking economy is that walking on treadmill requires more metabolic demands compared with that of overground walking. Therefore, those improvements in the treadmill group could be the result of a higher training intensity in comparison with the overground group. It can be argued that the use of the treadmill preferred speed rather than the overground preferred speed would be more ideal in order to match the energy cost between both surfaces. However, Dal et al. (20) reported that although the preferred walking speed in healthy subjects is lower on the treadmill, the oxygen uptake is higher when walking at the preferred treadmill speed compared with the preferred overground speed. We decided to use the overground preferred speed on the treadmill since this speed has been shown to induce gait improvements in PD (17, 29).

An alternative explanation for the more efficient walking gait pattern observed in PD, as a result of treadmill training, is that there was an improvement in the stride length of the patients. It has been shown that PD patients are unable to internally generate sufficiently large steps (2), thus leading to a higher cadence during slow to medium walking speeds in order to compensate the reduced stride length (2). This alteration in the normal stride length/cadence may contribute to the ameliorated walking economy reported in PD patients (5). In healthy subjects, a combination of short stride length/high cadence results in an increase of 18% in oxygen uptake in comparison with the preferred step cadence (30). A previous report showed that the patients from the treadmill group improved their stride length when walking at their preferred speed (12). This may reflect a new stride length/cadence relationship and a change from a lesser to a more efficient gait pattern as a result of the treadmill program.

A recent study (11) indicated that in PD patients, a low intensity treadmill training program is more efficient in improving gait compared with a high intensity one. Based on these findings, the authors point out that “*results based on treadmill training cannot be applied to overground walking without further study*” (11). Our results helps to clarify this issue suggesting that in mild PD patients, training on a treadmill is more beneficial than overground. More studies are needed in order to generalize these findings to the entire spectrum of PD patients.

In summary, the current study shows that a treadmill, but not an overground, walking training program of low volume and low intensity induces an improvement in the walking economy of PD. In addition, walking on a treadmill requires higher metabolic demands compared with overground walking at the same speed. These findings are of clinical importance when prescribing exercise in PD patients, in order to reduce the fatigue associated with the exercise itself.

## Conflict of Interest Statement

The authors declare that the research was conducted in the absence of any commercial or financial relationships that could be construed as a potential conflict of interest.
